# The anticoagulant effects of ethyl pyruvate in whole blood samples

**DOI:** 10.1371/journal.pone.0240541

**Published:** 2020-10-09

**Authors:** Harald Haidl, Axel Schlagenhauf, Angelika Krebs, Harald Plank, Willibald Wonisch, Vera Fengler, August Fiegl, Gerd Hörl, Martin Koestenberger, Thomas Wagner, Erwin Tafeit, Gerhard Cvirn, Seth Hallström

**Affiliations:** 1 Department of Pediatrics, Medical University of Graz, Graz, Austria; 2 Center for Medical Research, Medical University of Graz, Graz, Austria; 3 Institute for Electron Microscopy and Nanoanalysis, Graz University of Technology, Graz, Austria; 4 Division of Physiological Chemistry, Otto Loewi Research Center, Medical University of Graz, Graz, Austria; 5 Department of Blood Group Serology and Transfusion Medicine, Medical University of Graz, Graz, Austria; University of South Carolina, UNITED STATES

## Abstract

**Background:**

Ethyl pyruvate (EP), the ethyl ester of pyruvate, has proven antiinflammatory and antioxidative properties. Additionally, anticoagulant properties have been suggested recently. EP, therefore, is a potentially antiatherosclerotic drug. We aimed to investigate whether EP possesses antiplatelet and anticoagulant properties particularly in the physiological environment of whole blood.

**Methods:**

We investigated the effects of increasing concentrations of EP on platelet function, on the course of clot development, and on standard coagulation times. Additionally, clot ultrastructure using scanning electron microscopy was analysed.

**Results:**

EP exerted significant antiplatelet actions: i) Impedance aggregometry amplitudes (11.7 ± 3.0 ohm, 0 μg/mL EP) dose dependently decreased (7.8 ± 3.1 ohm, 1000 μg/mL EP; -33.3%). ATP exocytosis (0.87 ± 0.24 nM, 0 μg/mL EP) measured by the luminiscent method dose-dependently decreased (0.56 ± 0.14 nM, 1000 μg/mL; -35.6%). ii) Closure times (104.4 ± 23.8 s, 0 μg/mL EP) using the Platelet function analyzer were dose-dependently prolonged (180.5 ± 82.5 s, 1000 μg/mL EP; +72.9%) using membranes coated with collagen/ADP. iii) Surface coverage (15.9 ± 5.1%, 0 μg/mL EP) dose-dependently decreased (9.0 ± 3.7%, 1000 μg/mL EP; -43.4%) using the Cone and Platelet analyzer. EP also exerted significant anticoagulant actions: Coagulation times (177.9 ± 37.8, 0 μg/mL EP) evaluated by means of thrombelastometry were dose-dependently prolonged (212.8 ± 57.7 s, 1000 μg/mL EP; +19.6%). Activated partial thromboplastin times (31.5 ± 1.8 s, 0 μg/mL EP) were dose-dependently prolonged (35.6 ± 2.3 s, 1000 μg/mL EP; +13.0%). Prothrombin times (0.94 ± 0.02 INR, 0 μg/mL EP) were dose-dependently prolonged (1.09 ± 0.04 INR, 1000 μg/mL EP; +16.0%).

**Conclusion:**

We found that EP possesses antiplatelet and anticoagulant properties in whole blood. Together with its proven anti-inflammatory and antioxidative properties, EP is a potentially antiatherogenic drug.

## Introduction

Ethyl pyruvate (EP), the ethyl ester of pyruvate, is a compound with proven combined antiinflammatory and antioxidative effects [1–3]. Thus, it has been suggested that administration of EP can provide therapeutic benefits in many clinical situations, such as sepsis/inflammation, haemorrhage or atherosclerosis [4, 5]. For example, EP has been shown to be capable of attenuating the oxidation of low-density lipoprotein (LDL), a crucial step in the development of atherosclerosis [6].

Several recent studies have demonstrated that EP also exerts anticoagulant effects. For example: EP dose-dependently inhibits the expression and function of tissue factor (TF), the main initiator of coagulation activation in lipopolysaccharide (LPS)-stimulated monocytes [7] or is also capable to inhibit platelet aggregation [8].

However, most of the previous studies dealing with the effects of EP on the coagulation system were not performed in humans but in animal models, in cell culture or in purified systems containing washed platelets.

It was therefore the aim of our study to mainly examine the anticoagulant effects of EP in whole blood (WB) samples of human origin containing all coagulation factors, monocytes and platelets implicated in the coagulation process.

We investigated the effects of increasing concentrations of EP (0 up to 1000 μg/mL) on platelet aggregation (impedance method), on the course of clot development (thrombelastometry, TEM), on platelet function (platelet function analyser, PFA 200), and on platelet adhesion (cone and platelet analyser, CPA) in WB samples. Additionally, standard coagulation times (activated partial thromboplastin time, APTT; prothrombin time, PT) and thrombin generation curves (calibrated automated thrombogram, CAT) were measured in platelet poor plasma (PPP) samples.

Particularly, when examining the effects of increasing concentrations of EP on clot development and on thrombin generation curves, low amounts of TF were used in our experiments to trigger the coagulation process. This allows a highly sensitive and close to the in-vivo situation assessment of how increasing concentrations of EP may affect coagulation values [9]. Furthermore the influence of EP on exocytosis of ADP and ATP was examined by using HPLC [10] or a luciferase test. Additionally, the effect of EP on clot ultrastructure was studied by scanning electron microscopy (SEM) [11].

EP with its known anti-inflammatory and antioxidative properties together with antiplatelet and antithrombotic effects studied here in WB may further implicate its possible benefit as a drug in the setting of atherosclerosis, a disease well-known to be associated with inflammation, oxidative stress and activation of the coagulation cascade [12, 13].

## Material and methods

### Subjects

After approval of the appropriate institutional ethics committee (Ethikkommission der Medizinischen Universität Graz) and with written informed consent, a total of 43 healthy male volunteers aged between 27 and 47 years were recruited. This study was conducted in accordance with the Declaration of Helsinki. All volunteers denied taking any medication which might influence coagulation within the last two weeks. The volunteers did not suffer from renal or liver disease or coagulation disorders.

### Blood collection and preparation

A tube containing EDTA was first sampled in order to determine full blood count and to exclude initial coagulation activation from venepuncture and venous stasis. Subsequently, 9 mL of blood from the antecubital vein were collected into pre-citrated Vacuette^®^ marked tubes (Greiner Bio-one GmbH, Kremsmünster, Austria) containing 3.8% sodium citrate.

WB measurements (impedance aggregometry, TEM, platelet function tests) were performed within 3h of blood sampling. The remaining WB was centrifuged (room temperature, 20 min, 500g) in order to prepare PPP samples. Standard coagulation times and the time-course of thrombin generation were evaluated in PPP samples.

### Reagents

Purified EP was purchased from Sigma-Aldrich Handels GmbH (Vienna, Austria). A stock solution was prepared by 1:50 dilution in PBS. EP levels were successively raised (0, 250, 500, and 1000 μg/mL; this is equivalent to 0, 2, 4, and 8 mmol/L) by addition of increasing amounts of purified EP stock solution (0–50 μL) to 1 mL of WB, PRP or PPP. Innovin^®^ (recombinant human TF thromboplastin) from Dade Behring Marburg GmbH (Marburg, Germany), was used as a source of TF. The lyophilized product was dissolved in 10 mL of distilled water and subsequently diluted at a ratio of 1:1000 in 0.9% saline solution (TF-stock solution).

### Whole blood platelet aggregation assay

WB aggregation assessments were performed using a Chrono-Log Whole Blood Aggregometer Model 590 from Probe & Go (Endingen, Germany), which is based on the impedance method [14]. Impedance aggregometry results are expressed as amplitude (or maximum aggregation) in ohm at six minutes after reagent addition and as lag time (or aggregation time) in seconds, the time interval until the onset of platelet aggregation. The rate of platelet aggregation is expressed as slope in ohm/min. Collagen (2 μg/mL, final concentration), purchased from Probe & Go (Endingen, Germany), was used as platelet agonist, as previously described [15]. ATP release, a measure for the extent of the aggregation provides evidence of normal or impaired release from dense granule, was measured by a sensitive luminescent (firefly luciferin-luciferase) assay for extracellular ATP [16]. Additionally, the aggregability of platelets was assessed by quantitative determination of ATP and ADP exocytosis in platelet rich plasma (PRP) samples using a HPLC method [17]. WB samples were centrifuged at 150g for 12 min in order to obtain PRP samples. Platelet aggregation was induced by addition of collagen (2 μg/mL, final concentration) to 500 μL of the PRP samples. After two minutes, the PRP samples were centrifuged at 1500g for two minutes and proteins in the supernatant were precipitated with 200 μL 0.4 mol/L perchloric acid. After centrifugation at 12000g 100 μL of the supernatant were neutralized by addition of 10–12 μL of 2 mol/L K_2_CO_3_ at 4°C. The supernatant obtained after centrifugation was used for HPLC analysis (injection volume: 40 μL). Separation of adenine nucleotides was performed on a Hypersil ODS column (5 μm, 250 × 4 mm I.D., equipped with a precolumn; Thermo Electron Corp. Runcorn, Cheshire, UK) using a L-2200 autosampler, two L-2130 HTA pumps, and a L-2450 diode array detector (all VWR International, West Chester, PA, USA) as previously described [18]. Detector signals (absorbance at 254 nm) were recorded and the program EZchrom Elite (VWR) was used for data acquisition and analysis.

### Whole blood tissue factor-triggered TEM assay

The clot formation process was monitored using the TEM coagulation analyser (ROTEM^®^05) from Matel Medizintechnik (Graz, Austria). The period of time from adding trigger to initial fibrin formation is designated as the ‘Coagulation time’ (CT); the time until the amplitude reaches 20 mm refers to the ‘Clot formation time’ (CFT). ‘Maximum clot firmness’ (MCF) reflects clot stability and the ‘alpha angle’ indicates the velocity of fibrin built-up and cross-linking. The final sample volume was 340 μL. Clot formation was initiated by addition of 40 μL of ‘trigger solution’ containing TF and CaCl_2_ (0.35 pmol/L and 3 mmol/L final concentration, respectively) to 300 μL of citrated WB. This method has been described in detail previously by Sorensen et al. [19].

### Evaluation of primary haemostasis

Using the PFA 200, primary haemostasis is simulated with an in vitro quantitative measurement of platelet adhesion and aggregation in WB. The system uses citrated WB (800 μL) that is aspired under high shear stress rates through an aperture cut into a membrane coated with collagen (a subendothelial protein generally believed to be the initial matrix for platelet attachment) and either ADP or epinephrine. In response to the local shear stress and the agonists in the membrane, platelets are activated, adhere to collagen in the membrane surrounding the aperture, and aggregate until a stable platelet plug occludes the blood flow through the aperture. This time period recorded by the instrument is designated as the closure time, representing a measure of platelet-dependent haemostasis, in particular platelet activation, adherence, and aggregability [20].

### Whole blood platelet adhesion assay

Platelet adhesion and aggregation was assessed by using a CPA (DiaMed, Linz, Austria) as described previously [21]. Briefly, 130 μL of citrated WB was placed in polystyrene tubes and allowed to flow (1300 s^-1^) for two minutes using a rotating Teflon cone. Subsequently, the wells were washed with PBS, stained with May-Grünwald solution and analyzed with an image analysis system. Surface coverage (SC) and average size (AS) were determined to elucidate platelet function. SC, representing platelet adhesion, is expressed as the percentage of total area covered by platelets. AS, representing platelet aggregation, is defined as the average size of the surface bound objects.

### Automated fluorogenic measurement of thrombin generation

Thrombin generation curves were monitored using calibrated automated thrombography (CAT) (Thrombinoscope BV, Maastricht, the Netherlands) [22]. The ability of a plasma sample to generate thrombin was assessed with respect to lag time preceding the thrombin burst (Lag Time), time to peak (ttPeak), peak height (Peak), maximum velocity of thrombin formation (VelIndex) and endogenous thrombin potential (ETP), and the time point of free thrombin disappearance (StartTail). Measurements were carried out in the presence of five pM of TF (final concentration). Measuring the formation of thrombin, the pivotal enzyme in haemostasis, has been shown to be an appropriate method to assess the coagulability of a given plasma sample [22, 23].

### Standard coagulation tests

Haematocrit and blood cell counts were determined on a Sysmex KX-21 N Automated Haematology Analyzer from Sysmex (Illinois, USA). Determinations of PTs, APTTs as well as of plasma levels of FII, FVII, and FVIII, were performed on a BM/Hitachi 917 from Roche (Vienna, Austria).

### Scanning electron microscopy (SEM)

WB was centrifuged (room temperature, 12 min, 150g) in order to prepare PRP samples containing approximately 100 000 platelets/μL. Clot formation was initiated by addition of 40 μL of ‘trigger solution’ containing TF and CaCl_2_ (3.5 pmol/L and 3 mmol/L final concentration, respectively) to 300 μL of PRP. After overnight clot formation, the clot ultrastructure of unfixed and carbon coated specimen was characterized via SEM, performed in a NOVA 200 dual beam microscope (Thermo fisher, USA). To minimize electron-beam related damage, low primary energies (5 keV) and beam currents (98 pA) were used with shortest possible image acquisition times [24].

### Statistics

Statistical analyses were performed using the GraphPad 7.0 Prism package. The effects of increasing concentrations of EP on coagulation values were analysed using linear regression, expressed as slope ± standard error [25]. All p-values of <0.05 were considered statistically significant. Further statistical calculations were performed by IBM SPSS Statistics (version 26). The normal distribution of the variables was tested using the Shapiro-Wilk test and the Kolmogorov-Smirnov test. Differences in the distributions of variables with differing EP concentrations were tested by a paired-samples t-test (in case of normally distributed variables) and by a non-parametric Wilcoxon test (if variables were not normally distributed) [26]. *… p<0.05, **… p<0.01, ***… p<0.001.

## Results

Blood cell counts and plasma levels of FII, FVII, and FVIII were within the normal ranges for adults [15, 27]. EP levels in WB, PRP or PPP samples were raised from 0 to 250, 500, or 1000 μg/mL (final concentration), which is equivalent to 0, 2, 4, and 8 mmol/L (final concentration), by addition of increasing amounts of purified EP stock solution.

### Effects of increasing concentrations of EP on impedance aggregometry values

**Fig 1:**
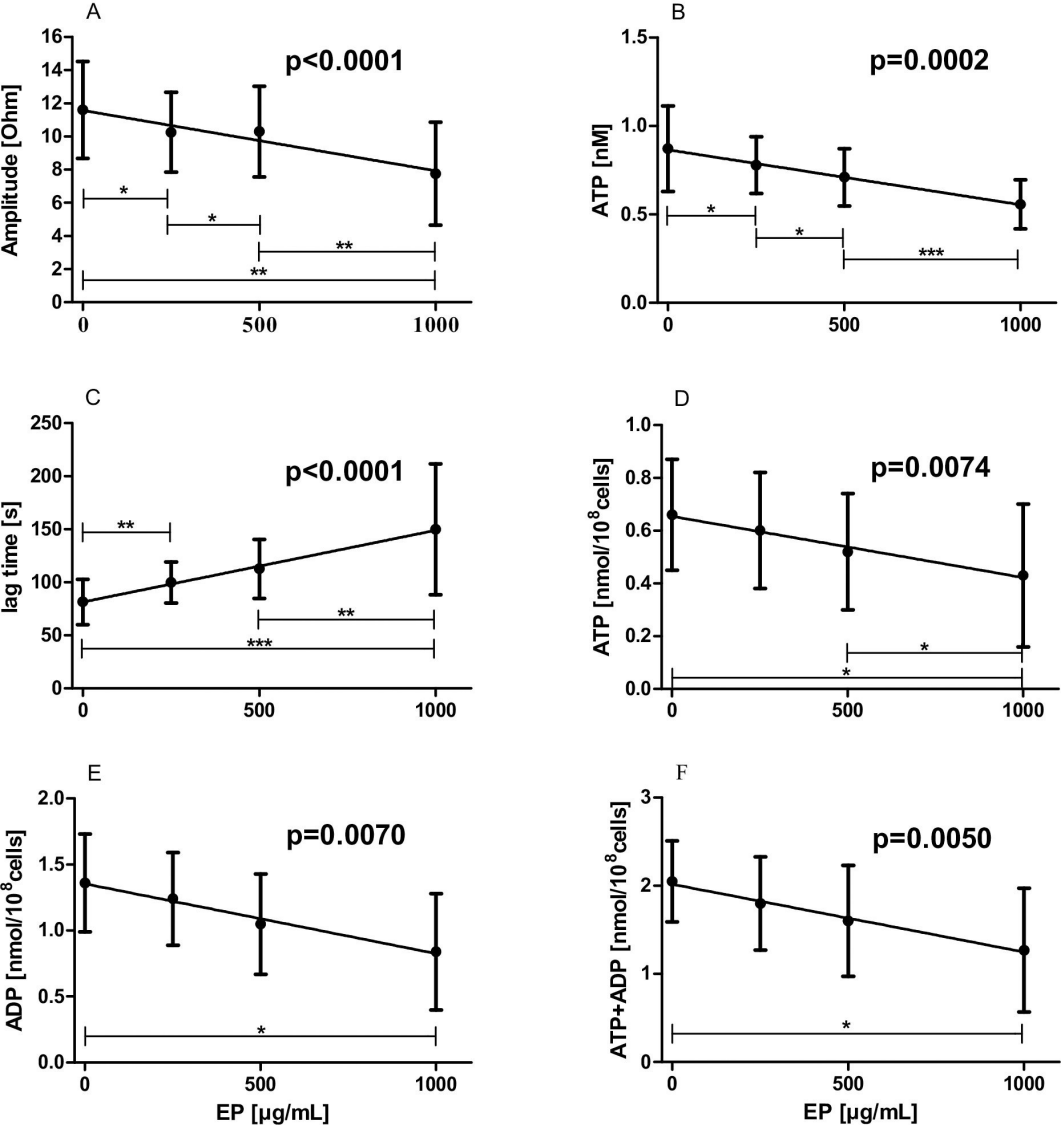
Effects of increasing concentrations of EP on impedance aggregometry values. WB samples (n = 35) or PRP samples (n = 6) were pre-incubated with 0 up to 1000 μg/mL EP for 5 min. Subsequently, platelet aggregation was triggered by addition of collagen (2 μg/mL final concentration). (A) Amplitudes; (B), exocytosis of ATP in WB; (C) Lag times; (D) ATP exocytosis in PRP; (E) ADP exocytosis in PRP; (F) sum of these co-released nucleotides are illustrated. Data represent mean ± SD.

Significant antiplatelet effects of EP were observed by means of impedance aggregometry performed in WB samples. In the presence of increasing concentrations of EP amplitudes and ATP exocytosis measured by the luminescent method dose-dependently decreased (amplitudes: slope: -0.003677 ± 0.000766 ohm·mL/μg, p<0.0001; ATP exocytosis: slope: -0.000312 ± 0.000076 nM·mL/μg, p = 0.0002, respectively), shown in Fig 1A and 1B, respectively. Lag times were dose-dependently prolonged by increasing EP concentrations (slope: 0.067689 ± 0.009713 min·mL/μg, p<0.0001; Fig 1C). Slopes were not altered by increasing EP concentrations (-0.001369 ± 0.000635 ohm·mL/min·μg, p = 0.1637).

Measurement of adenine nucleotides in six PRP samples by HPLC revealed commensurable results as obtained in WB samples using the luminescent method. ATP reduction as well as declining ADP exocytosis from dense granules due to increasing concentrations of EP (ATP: slope: -0.000232 ± 0.000020 nmol·mL/μg·10^8^cells, *p* = 0.0074, Fig 1D; ADP: slope: -0.000530 ± 0.000044 nmol·mL/μg·10^8^cells, p = 0.0070, Fig 1E) were observed. This is also demonstrated by the sum of these co-released adenine nucleotides (ATP + ADP: slope: -0.000768 ± 0.000054 nmol·mL/μg·10^8^cells, p = 0.0050, Fig 1F).

### Effects of increasing concentrations of EP on TEM values

**Fig 2:**
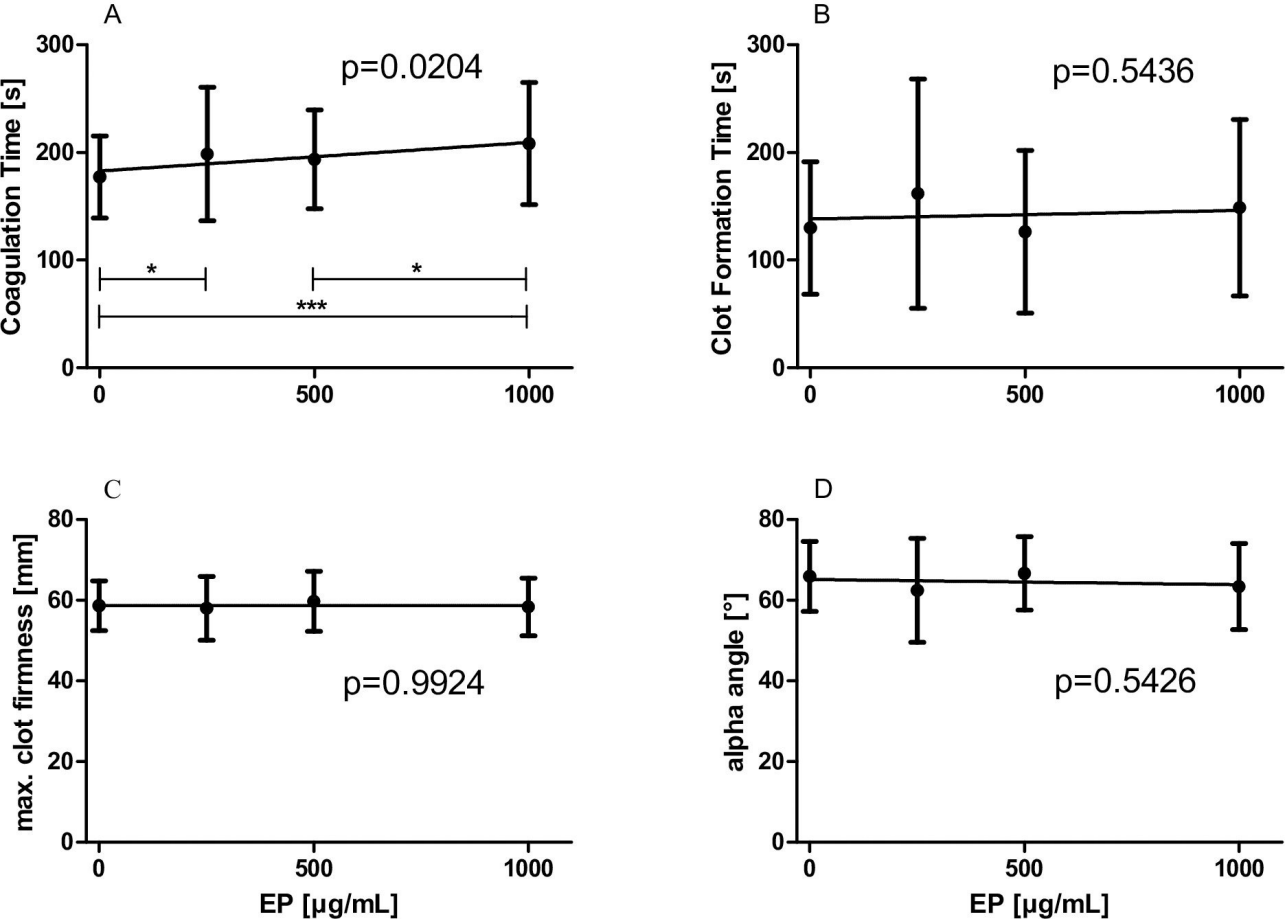
Effects of increasing concentrations of EP on TEM values. WB samples (n = 42) were pre-incubated with 0 up to 1000 μg/mL EP for 5 min. Subsequently, clot formation was triggered by addition of TF/CaCl_2_ (0.35 pmol/L and 3 mmol/L final concentration, respectively). (A) Coagulation times; (B) Clot formation times; (C) Maximum clot firmness; (D) alpha angles are illustrated. Data represent mean ± SD.

CTs were dose-dependently prolonged in the presence of increasing concentrations of EP, shown in Fig 2A (slope: 0.02928 ± 0.012435 s·mL/μg, p = 0.0204). The remaining TEM values were not changed by increasing EP concentrations as shown in Fig 2. CFT: slope: 0.012215 ± 0.020045 s·mL/μg, p = 0.5436, Fig 2B; MCF: slope: 0.000017 ± 0.001788 mm·mL/μg, p = 0.9924, Fig 2C; alpha: slope: -0.001517 v 0.002484 [°]·mL/μg, p = 0.5426, Fig 2D.

### Effects of increasing concentrations of EP on platelet function (using PFA 200)

**Fig 3:**
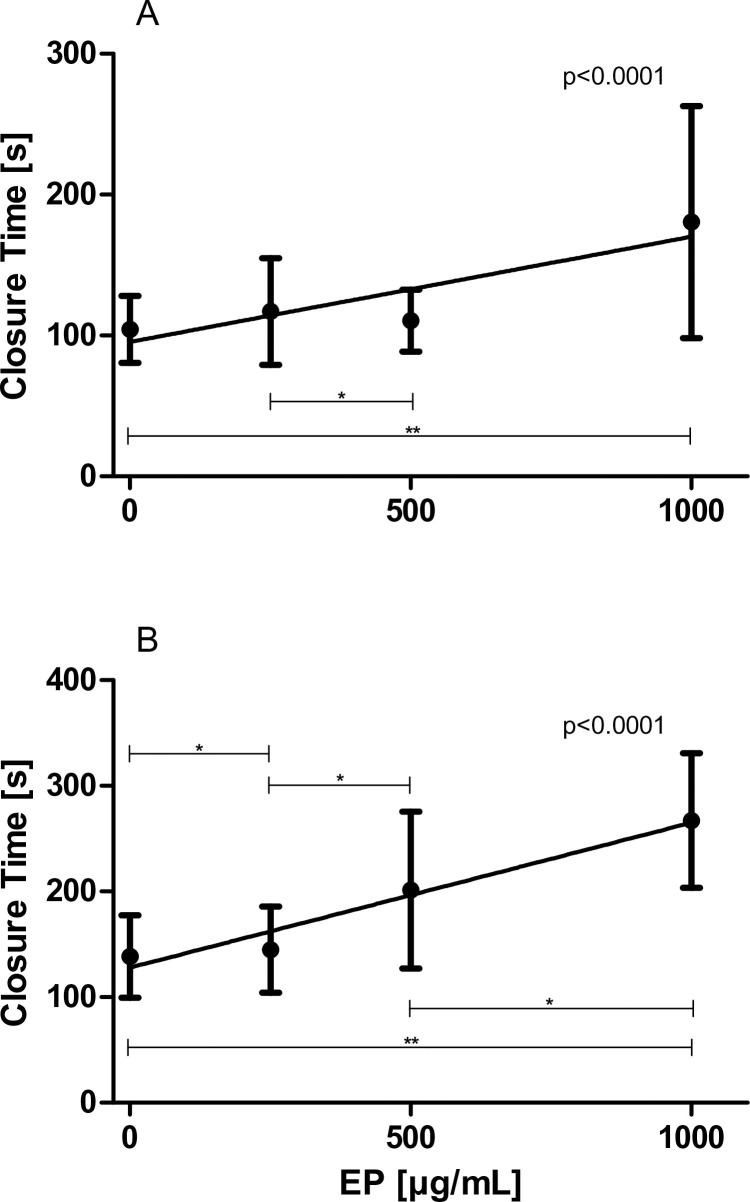
Effects of increasing concentrations of EP on primary haemostasis (PFA 200). WB samples (n = 28) were pre-incubated with 0 up to 1000 μg/mL EP for 5 min. (A) Closure times using membranes coated with collagen/ADP and (B) Closure times using membranes coated with Collagen/epinephrine are illustrated. Data represent mean ± SD.

EP significantly blunted platelet function (i.e., platelet adhesion and aggregation) in WB samples using the PFA 200 device. Closure times were dose-dependently prolonged in the presence of increasing concentrations of EP utilizing membranes coated with collagen/ADP (slope: 0.068894 ± 0.014342 s·mL/μg, p<0.0001; Fig 3A) as well as utilizing membranes coated with collagen/epinephrine (slope: 0.132049 ± 0.019182 s·mL/μg, p<0.0001; Fig 3B).

### Effects of increasing concentrations of EP on platelet adhesion (using CPA)

**Fig 4:**
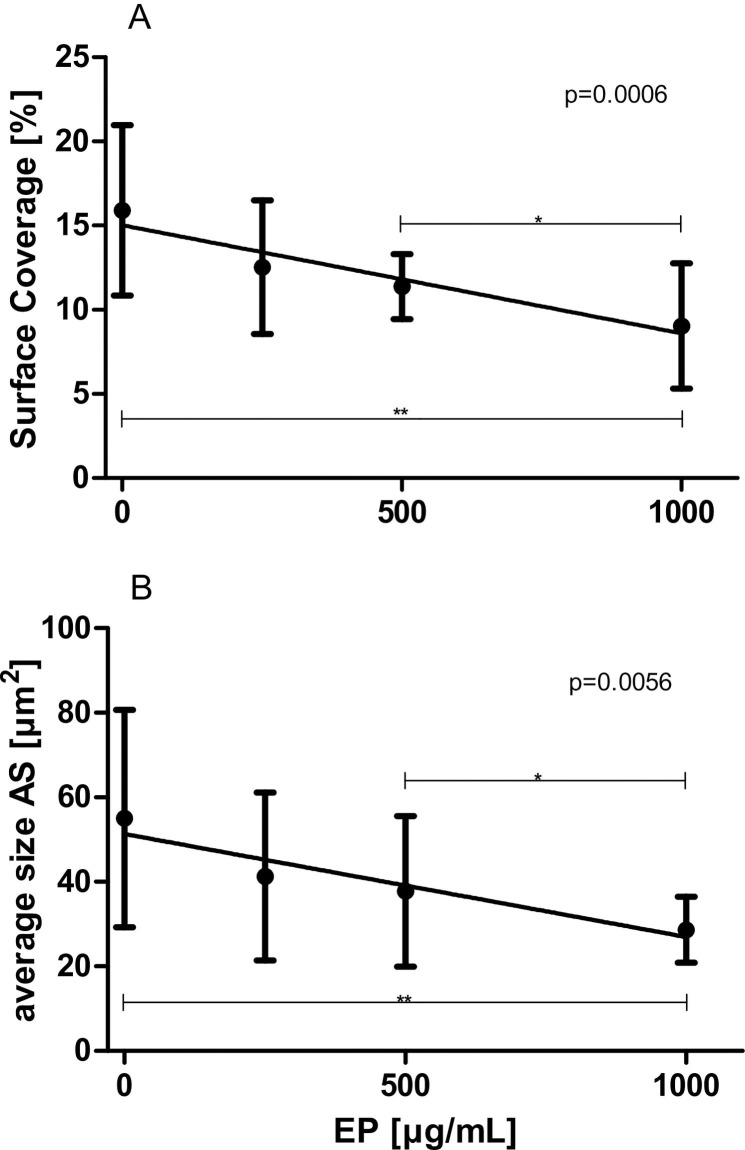
Effects of increasing concentrations of EP on platelet adhesion and aggregation (CPA). WB samples (n = 10) were pre-incubated with 0 up to 1000 μg/mL EP for 5 min. (A) Surface coverage and (B) Average size are illustrated. Data represent mean ± SD.

EP significantly attenuated platelet adhesion in WB samples using the PFA 200 device. Both SC (Fig 4A) as well as AS (Fig 4B) dose-dependently decreased in the presence of increasing concentrations of EP (SC: slope: -0.006526 ± 0.001720%·mL/μg, p = 0.0006; AS: slope: -0.024797 ± 0.008374 μm^2^·mL/μg, p = 0.0056, respectively).

### Effects of increasing concentrations of EP on standard coagulation times

**Fig 5:**
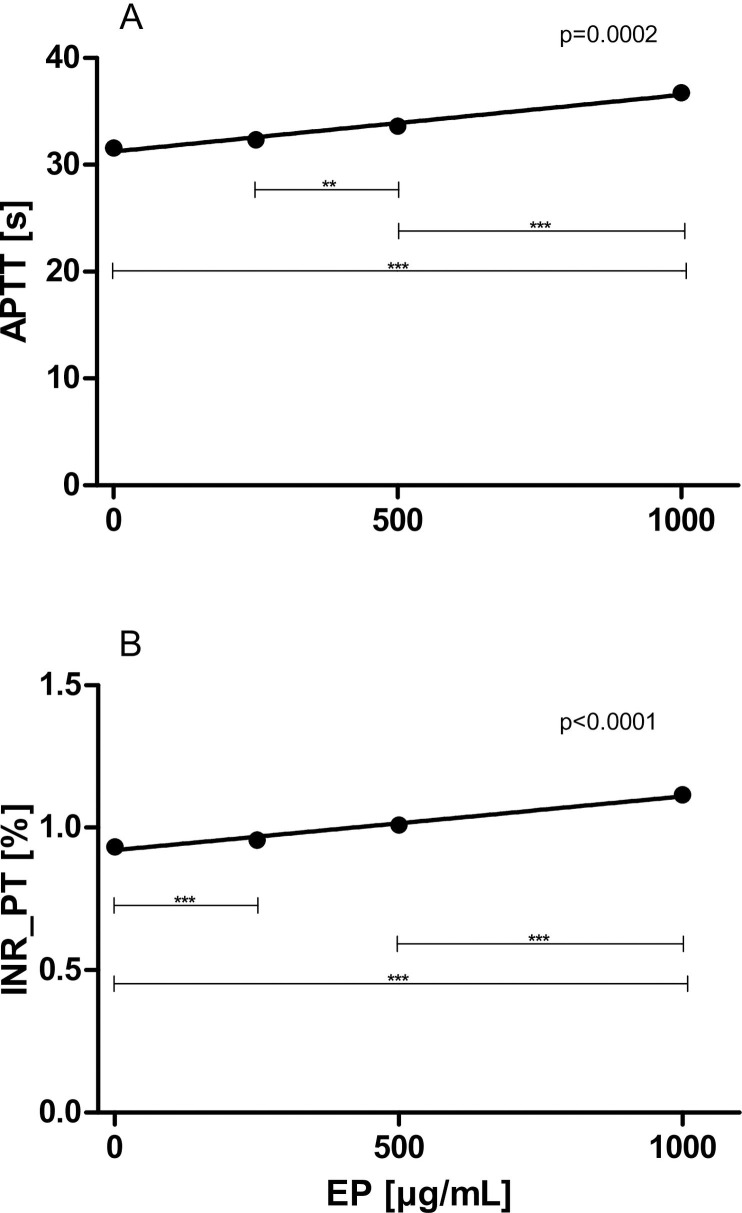
Effects of increasing concentrations of EP on standard coagulation times. PPP samples (n = 8) were pre-incubated with 0 up to 1000 μg/mL EP for 5 min. (A) APTTs and (B) PTs are illustrated. Data represent mean ± SD.

EP exerted significant anticoagulant action, shown in Fig 5. Both APTTs as well as PTs (expressed as international normalized ratio INR) were dose-dependently prolonged in the presence of increasing concentrations of EP (APTT: slope: 0.004296 ± 0.000999 s·mL/μg, p = 0.0002, Fig 5A; PT: slope: 0.000150 ± 0.000013%·mL/μg, p<0.0001, Fig 5B).

### Effects of increasing concentrations of EP on thrombin generation (CAT)

**Fig 6:**
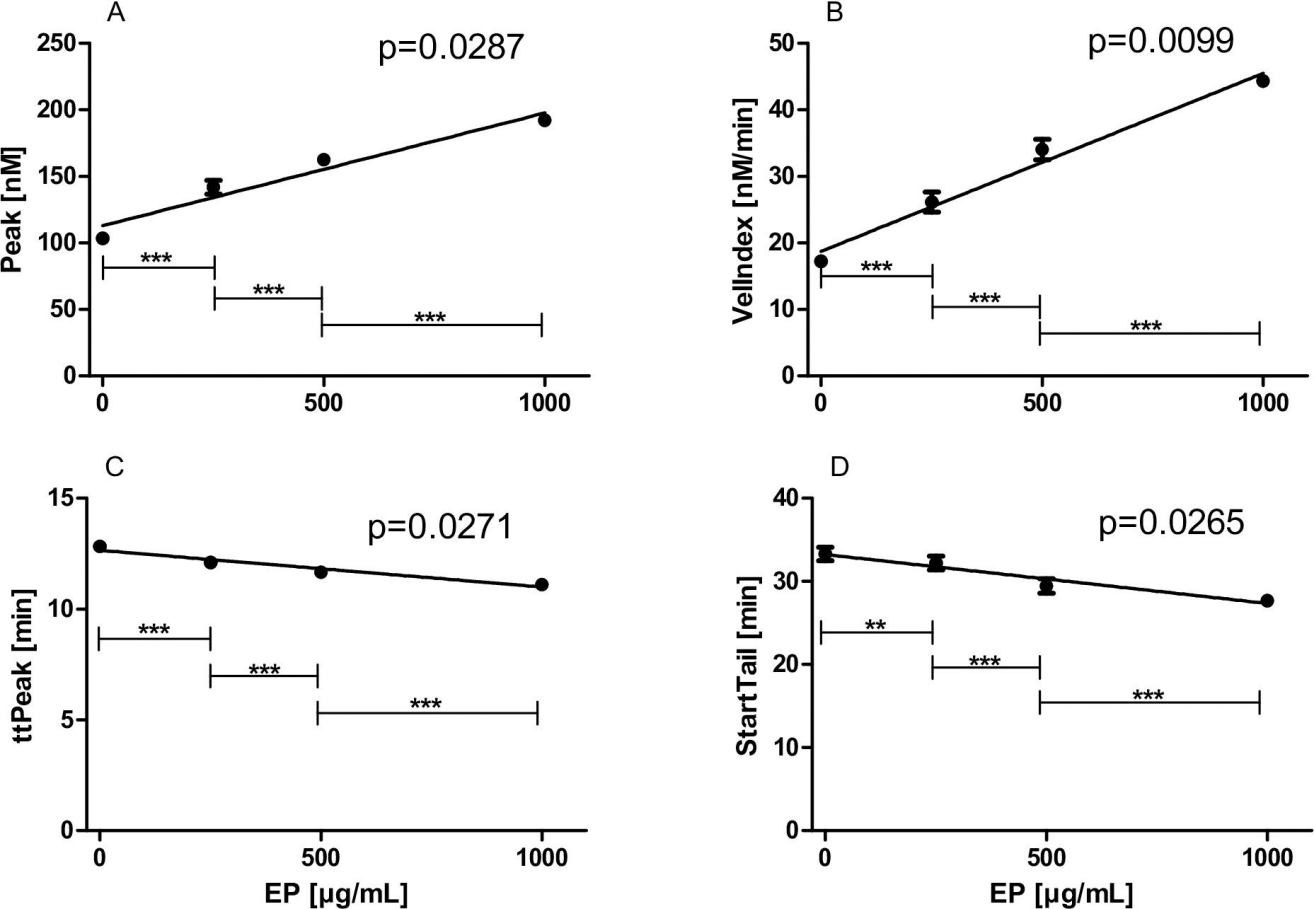
Effects of increasing concentrations of EP on thrombin generation (CAT). PPP samples (n = 10) were pre-incubated with 0 up to 1000 μg/mL EP for 5 min. (A) Peak; (B) VelIndex; (C) ttPeak; and (D) StartTail are illustrated. Data represent mean ± SD.

Lag times (slope: 0.000010 ± 0.000154, p = 0.9529) and ETP (slope: 0.2267 ± 0.1302, p = 0.2237) were not changed by different EP concentrations. However, four CAT values indicated increasing thrombin generation in the presence of increasing concentrations of EP, shown in Fig 6. Peak (slope: 0.084850 ± 0.01469 nM·mL/μg, p = 0.0287, Fig 6A) and VelIndex (slope: 0.026780 ± 0.002683 nM·mL/min·μg, p = 0.0099, Fig 6B) dose-dependently increased and ttPeak (slope: -0.001655 ± 0.000278 min·mL/μg, p = 0.0271, Fig 6C) and StartTail (slope: -0.005886 ± 0.000978 min·mL/μg, p = 0.0265, Fig 6D) dose-dependently decreased in the presence of increasing concentrations of EP.

### Effects of EP on clot ultrastructure (SEM)

**Fig 7:**
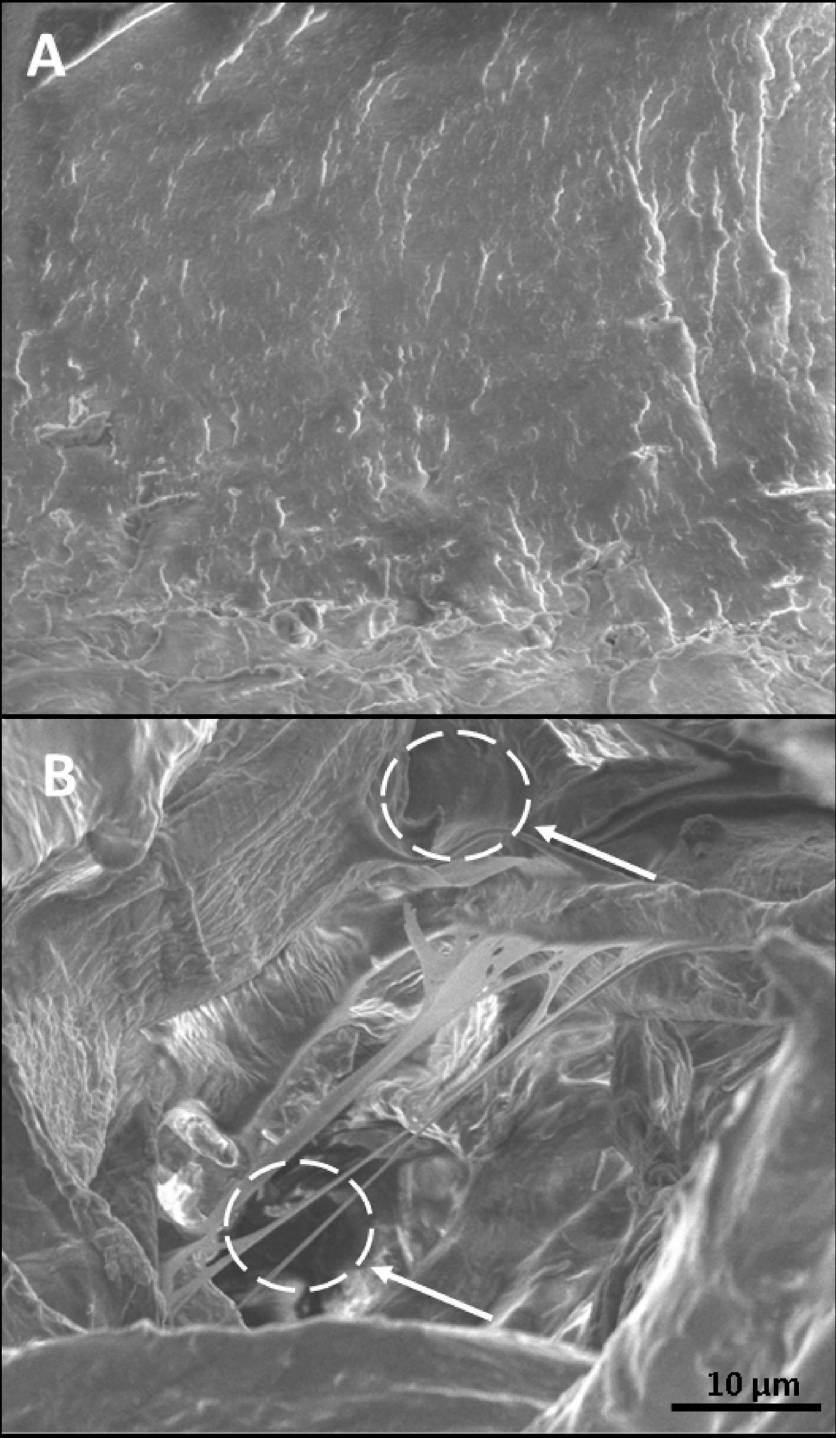
Effects of EP on the clot ultrastructure evaluated by means of SEM. Clot formation was induced by addition of TF/CaCl_2_ (3.5 pmol/L and 3 mmol/L final concentration, respectively) in the (A) absence or (B) presence of 1000 μg/mL EP. Clots from three individuals were analysed, a representative experiment is shown. An intrinsic pore is marked in (B).

In the absence of EP a compact clot was formed when PRP (100 000 platelet/μL) was incubated with an initiator solution containing TF/CaCl_2_ (3.5 pmol/L and 3 mmol/L final concentration). When clot formation was initiated in the presence of 1000 μg/mL EP, the clot structure was less compact and contained intrinsic pores at sizes between 3 and 5 μm (Fig 7).

## Discussion

The present study indicates that EP is a potentially antiplatelet and anticoagulant compound. To the best of our knowledge, by utilizing impedance aggregometry, we for the first time show that EP dose-dependently inhibits platelet aggregation and ATP release in the physiological environment of WB. We observed a delayed onset and a decreased maximum aggregation, associated with decreased ATP exocytosis (fluorescent method), in the presence of increasing amounts of EP. This is in principal in good agreement with results from a previous study in which PRP or washed platelets of human and murine origin were used [8]. Whereby, choosing WB samples for platelet aggregation analysis apparently better reflects the in-vivo situation than PRP/washed platelets. WB not only contains a majority of the coagulation factors implicated in the coagulation process but also phospholipid bearing cells, e.g., leukocytes, which, by interacting with platelets, have been shown to be of importance during coagulation and fibrinolysis [28–30]. We additionally studied the release of adenine nucleotides in six PRP samples under increasing concentrations of EP by an HPLC method. In good agreement with the results from the luminescent method, we found that ATP as well as ADP release dose-dependently decreased with increasing concentrations of EP. The two extracellular signalling molecules ATP and ADP interact with the platelet P2 receptors to amplify ongoing platelet activation. Recent advances in the understanding of the P2Y receptor physiology have reinforced the concept of these receptors as useful targets for antithrombotic therapy [31]. Thus, EP might be a suitable drug to attenuate ATP/ADP-induced platelet activation.

In addition to the impedance aggregometry results, both PFA 200 and CPA measurements also indicated significant antiaggregatory actions of EP: Closure times (evaluated by means of the PFA 200) were dose-dependently prolonged and AS (evaluated by means of a CPA) dose-dependently decreased in the presence of increasing concentrations of EP.

The present study also indicates that EP is capable of inhibiting platelet adhesion in WB samples: the SC, examined by means of a CPA, dose-dependently decreased in the presence of increasing concentrations of EP. This, again, is in good agreement with the study performed by Li et al. [8]. Using washed platelets preincubated with increasing amounts of EP, they also observed that platelet adhesion, i.e., SC, was decreased by EP. They have shown that EP is capable of inhibiting phosphatidylinositol 3-kinase/Akt and protein kinase signalling, common pathways involved in platelet activation.

Administration of EP might be more suitable for the prevention and therapy of coronary and other atherosclerotic vascular diseases than aspirin, the most extensively studied and administered antiplatelet agent: In contrast to aspirin, EP is capable of attenuating the expression of platelet activation markers, i.e. CD40L [32]. This marker has been shown to promote the recruitment of leukocytes to endothelial cells with subsequent inflammation, development of atherosclerosis, followed by thrombo-ischemic events leading to myocardial infarction and ischemic stroke [33–36]. Our study also indicates that EP is a potentially anticoagulant drug. For the first time we show that both APTTs and PTs, evaluated in PPP samples, were dose-dependently prolonged in the presence of increasing concentrations of EP.

Only a few studies exist to date dealing with the effects of EP on standard coagulation times [3, 7, 37]. These studies, performed in animal models or in monocytic cell culture, also indicate that EP is an anticoagulant drug: the effects of haemorrhage or of administration of either LPS or resuscitation fluid on the PT have been shown to be reversed by EP. This anticoagulant action of EP has been attributed to its capability to suppress the formation of TF, the main initiator of coagulation [37]. However, these findings cannot directly be transferred to our present study in which the effects of EP on standard coagulation times were examined in PPP samples lacking TF-producing cells.

To our knowledge no data at present exist reporting on the effects of EP on the clot formation process. Results from our SEM measurements revealed alterations in the three-dimensional construction of the clot apparently caused by EP. However, future studies are urgently required to elucidate the mechanisms by which EP affects the clot formation process. Commensurably, EP was capable of prolonging CTs, evaluated by means of TEM, indicating a decelerated clot formation process.

Noteworthy, results from our CAT measurements indicate that EP also possesses procoagulant properties. For example, the (thrombin) peak dose-dependently increased in the presence of increasing concentrations of EP. On the other hand, we found that EP had no effect on the ETP, indicating that EP does not alter the total amount of thrombin being formed in a given plasma sample [38]. Thus, the procoagulant activities of EP might be of minor physiological or clinical significance.

A further mechanism through which EP might exert anticoagulant action in our experiments is its capability to chelate Ca^2+^ ion. However, this effect becomes significant only in the presence of high concentrations of EP exceeding 1000 μg/mL [39]. In conclusion, our study shows that EP possesses, besides its well-known antiinflammatory and antioxidative properties, also antiplatelet and anticoagulant properties in WB.

However, in a clinical trial of patients undergoing surgery short-term administration of EP failed to improve the outcome. Furthermore, the inflammatory reactions usually accompanying cardiac surgery were not blunted by EP [40]. Therefore, chronic administration of EP (and not short-term administration) might be a more suitable tool for this pluripotent agent. Due to its antiinflammatory, antioxidative, antiplatelet, and anticoagulant properties EP is presumably capable of preventing the formation of atherosclerotic plaques on several levels. EP has been shown to be capable of i) ameliorating endothelial cell injury in various inflammatory conditions due to its anti-inflammatory properties [5], ii) blunting the oxidation of LDL due to its antioxidative properties [6], and, as shown in the present study, iii) attenuating platelet adhesion/aggregation and thereby preventing activation of the coagulation cascade in case of plaque disruption which can lead to the life-threatening formation of a blood clot.

In conclusion, this study demonstrates significant anticoagulant properties of EP in the physiological environment of WB. EP is apparently a pluripotent pharmaceutical agent which can provide therapeutic benefits in many clinical situations such as sepsis/inflammation, haemorrhage or atherosclerosis. It has to be stated that the anticoagulant action of EP might not be beneficial in some clinical situations, e.g., in patients with inherited bleeding disorders (hemophilia A and B, Willebrand disease), as well as in patients suffering trauma. Moreover, most effective anticoagulant action was observed in our study in the presence of 1000 μg/mL (~8 mM) of EP. However, no clinical data exist to date reporting whether these high plasma levels of EP can be reached and maintained chronically in the patients and whether untoward side effects are associated with such high EP levels. Furthermore, since EP might undergo spontaneous hydrolysis to form pyruvic acid and ethanol, the resultant chronic ethanol exposure could be considered a limitation [4].

## Supporting information

S1 Data Data availability. (XLSX)

## Abbreviations

ADP: adenosine diphosphate
APTT: activated partial thromboplastin time
AS: average size
ATP: adenosine triphosphate
CAT: calibrated automated thrombography
CPA: cone and platelet analyzer
CFT: clot formation time
CT: coagulation time
EDTA: ethylene-diamine-tetraacetic acid
EP: ethyl pyruvate
ETP: endogenous thrombin potential
HPLC: high performance liquid chromatography
INR: international normalized ratio
MCF: maximum clot firmness
LDL: low-density lipoprotein
LPS: lipopolysaccharide
PBS: phosphate buffered saline
PFA: platelet function analyser
PPP: platelet poor plasma
PRP: platelet rich plasma
PT: prothrombin time
SC: surface coverage
SD: standard deviation
SEM: scanning electron microscopy
TEM: thrombelastometry
TF: lipidated tissue factor
WB: whole blood

## References

[1] Ulloa L, Ochani M, Yang H, Tanovic M, Halperin D, Yang R, et al Ethyl pyruvate prevents lethality in mice with established lethal sepsis and systemic inflammation. Proc Natl Acad Sci U S A. 2002;99(19):12351–6. 10.1073/pnas.192222999 12209006 PMC129448

[2] Miyaji T, Hu X, Yuen PS, Muramatsu Y, Iyer S, Hewitt SM, et al Ethyl pyruvate decreases sepsis-induced acute renal failure and multiple organ damage in aged mice. Kidney Int. 2003;64(5):1620–31. 10.1046/j.1523-1755.2003.00268.x . 14531793

[3] Dong W, Cai B, Pena G, Pisarenko V, Vida G, Doucet D, et al Ethyl pyruvate prevents inflammatory responses and organ damage during resuscitation in porcine hemorrhage. Shock. 2010;34(2):205–13. 10.1097/SHK.0b013e3181cc0c63 19953001 PMC2891599

[4] Kao KK, Fink MP. The biochemical basis for the anti-inflammatory and cytoprotective actions of ethyl pyruvate and related compounds. Biochem Pharmacol. 2010;80(2):151–9. 10.1016/j.bcp.2010.03.007 . 20230800

[5] Crawford RS, Albadawi H, Atkins MD, Jones JJ, Conrad MF, Austen WG Jr., et al Postischemic treatment with ethyl pyruvate prevents adenosine triphosphate depletion, ameliorates inflammation, and decreases thrombosis in a murine model of hind-limb ischemia and reperfusion. J Trauma. 2011;70(1):103–10; discussion 10. 10.1097/TA.0b013e3182031ccb 21217488 PMC3056773

[6] Rossmann C, Nusshold C, Paar M, Ledinski G, Tafeit E, Koestenberger M, et al Ethyl pyruvate inhibits oxidation of LDL in vitro and attenuates oxLDL toxicity in EA.hy926 cells. PLoS One. 2018;13(1):e0191477 10.1371/journal.pone.0191477 29370236 PMC5784938

[7] van Zoelen MA, Bakhtiari K, Dessing MC, van't Veer C, Spek CA, Tanck M, et al Ethyl pyruvate exerts combined anti-inflammatory and anticoagulant effects on human monocytic cells. Thromb Haemost. 2006;96(6):789–93. . 17139374

[8] Li W, Yang X, Peng M, Li C, Mu G, Chen F. Inhibitory effects of ethyl pyruvate on platelet aggregation and phosphatidylserine exposure. Biochem Biophys Res Commun. 2017;487(3):560–6. 10.1016/j.bbrc.2017.04.087 . 28427942

[9] Cvirn G, Gallistl S, Rehak T, Jurgens G, Muntean W. Elevated thrombin-forming capacity of tissue factor-activated cord compared with adult plasma. J Thromb Haemost. 2003;1(8):1785–90. 10.1046/j.1538-7836.2003.00320.x . 12911594

[10] Hallstrom S, Gasser H, Neumayer C, Fugl A, Nanobashvili J, Jakubowski A, et al S-nitroso human serum albumin treatment reduces ischemia/reperfusion injury in skeletal muscle via nitric oxide release. Circulation. 2002;105(25):3032–8. 10.1161/01.cir.0000018745.11739.9b . 12081999

[11] Danilatos GD, Postle R. The environmental scanning electron microscope and its applications. Scan Electron Microsc. 1982;(Pt 1):1–16. . 7167743

[12] Esterbauer H, Gebicki J, Puhl H, Jurgens G. The role of lipid peroxidation and antioxidants in oxidative modification of LDL. Free Radic Biol Med. 1992;13(4):341–90. 10.1016/0891-5849(92)90181-f . 1398217

[13] Stocker R, Keaney JF Jr. Role of oxidative modifications in atherosclerosis. Physiol Rev. 2004;84(4):1381–478. 10.1152/physrev.00047.2003 . 15383655

[14] Morel-Kopp MC, Tan CW, Brighton TA, McRae S, Baker R, Tran H, et al Validation of whole blood impedance aggregometry as a new diagnostic tool for HIT: results of a large Australian study. Thromb Haemost. 2012;107(3):575–83. 10.1160/TH11-09-0631 . 22234599

[15] Lamprecht M, Moussalli H, Ledinski G, Leschnik B, Schlagenhauf A, Koestenberger M, et al Effects of a single bout of walking exercise on blood coagulation parameters in obese women. J Appl Physiol (1985). 2013;115(1):57–63. 10.1152/japplphysiol.00187.2013 . 23620490

[16] Fritsma GA, McGlasson DL. Whole Blood Platelet Aggregometry. Methods Mol Biol. 2017;1646:333–47. 10.1007/978-1-4939-7196-1_26 . 28804840

[17] von Papen M, Gambaryan S, Schutz C, Geiger J. Determination of ATP and ADP Secretion from Human and Mouse Platelets by an HPLC Assay. Transfus Med Hemother. 2013;40(2):109–16. 10.1159/000350294 23652982 PMC3638993

[18] Deak AT, Blass S, Khan MJ, Groschner LN, Waldeck-Weiermair M, Hallstrom S, et al IP3-mediated STIM1 oligomerization requires intact mitochondrial Ca2+ uptake. J Cell Sci. 2014;127(Pt 13):2944–55. 10.1242/jcs.149807 24806964 PMC4077590

[19] Sorensen B, Johansen P, Christiansen K, Woelke M, Ingerslev J. Whole blood coagulation thrombelastographic profiles employing minimal tissue factor activation. J Thromb Haemost. 2003;1(3):551–8. 10.1046/j.1538-7836.2003.00075.x . 12871465

[20] Chang YW, Liao CH, Day YJ. Platelet function analyzer (PFA-100) offers higher sensitivity and specificity than thromboelastography (TEG) in detection of platelet dysfunction. Acta Anaesthesiol Taiwan. 2009;47(3):110–7. 10.1016/s1875-4597(09)60036-9 . 19762300

[21] Varon D, Dardik R, Shenkman B, Kotev-Emeth S, Farzame N, Tamarin I, et al A new method for quantitative analysis of whole blood platelet interaction with extracellular matrix under flow conditions. Thromb Res. 1997;85(4):283–94. 10.1016/s0049-3848(97)00014-5 . 9062952

[22] Al Dieri R, de Laat B, Hemker HC. Thrombin generation: what have we learned? Blood Rev. 2012;26(5):197–203. 10.1016/j.blre.2012.06.001 . 22762893

[23] Mann KG, Orfeo T, Butenas S, Undas A, Brummel-Ziedins K. Blood coagulation dynamics in haemostasis. Hamostaseologie. 2009;29(1):7–16. 19151839 PMC3152749

[24] Joy DC, Pawley JB. High-resolution scanning electron microscopy. Ultramicroscopy. 1992;47(1–3):80–100. 10.1016/0304-3991(92)90186-n . 1481281

[25] Jurimae T, Sudi K, Jurimae J, Payerl D, Moller R, Tafeit E. Validity of optical device lipometer and bioelectric impedance analysis for body fat assessment in men and women. Coll Antropol. 2005;29(2):499–502. . 16417151

[26] Kruschitz R, Wallner-Liebmann SJ, Hamlin MJ, Moser M, Ludvik B, Schnedl WJ, et al PLoS One. 2013;8(8): e72002 10.1371/journal.pone.0072002 . 23991025 PMC3753354

[27] Andrew M, Vegh P, Johnston M, Bowker J, Ofosu F, Mitchell L. Maturation of the hemostatic system during childhood. Blood. 1992;80(8):1998–2005. . 1391957

[28] McEver RP. Adhesive interactions of leukocytes, platelets, and the vessel wall during hemostasis and inflammation. Thromb Haemost. 2001;86(3):746–56. . 11583304

[29] Totani L, Amore C, Di Santo A, Dell'Elba G, Piccoli A, Martelli N, et al Roflumilast inhibits leukocyte-platelet interactions and prevents the prothrombotic functions of polymorphonuclear leukocytes and monocytes. J Thromb Haemost. 2016;14(1):191–204. 10.1111/jth.13173 . 26484898

[30] Ghasemzadeh M, Hosseini E. Platelet-leukocyte crosstalk: Linking proinflammatory responses to procoagulant state. Thromb Res. 2013;131(3):191–7. 10.1016/j.thromres.2012.11.028 . 23260445

[31] Oury C, Toth-Zsamboki E, Vermylen J, Hoylaerts MF. The platelet ATP and ADP receptors. Curr Pharm Des. 2006;12(7):859–75. 10.2174/138161206776056029 . 16515502

[32] Hermann A, Rauch BH, Braun M, Schror K, Weber AA. Platelet CD40 ligand (CD40L)—subcellular localization, regulation of expression, and inhibition by clopidogrel. Platelets. 2001;12(2):74–82. 10.1080/09537100020031207 . 11297035

[33] Zuern CS, Lindemann S, Gawaz M. Platelet function and response to aspirin: gender-specific features and implications for female thrombotic risk and management. Semin Thromb Hemost. 2009;35(3):295–306. 10.1055/s-0029-1222608 . 19452405

[34] Heemskerk JW, Mattheij NJ, Cosemans JM. Platelet-based coagulation: different populations, different functions. J Thromb Haemost. 2013;11(1):2–16. 10.1111/jth.12045 . 23106920

[35] Thomas MR, Storey RF. The role of platelets in inflammation. Thromb Haemost. 2015;114(3):449–58. 10.1160/TH14-12-1067 . 26293514

[36] Gawaz M, Langer H, May AE. Platelets in inflammation and atherogenesis. J Clin Invest. 2005;115(12):3378–84. 10.1172/JCI27196 16322783 PMC1297269

[37] Kung CW, Lee YM, Yen MH. In vivo anticoagulant effect of ethyl pyruvate in endotoxemic rats. Thromb Res. 2011;127(6):582–8. 10.1016/j.thromres.2011.01.017 . 21396682

[38] Ten Cate H, Hemker HC. Thrombin Generation and Atherothrombosis: What Does the Evidence Indicate? J Am Heart Assoc. 2016;5(8). 10.1161/JAHA.116.003553 27503850 PMC5015283

[39] Shin JH, Lee HK, Lee HB, Jin Y, Lee JK. Ethyl pyruvate inhibits HMGB1 phosphorylation and secretion in activated microglia and in the postischemic brain. Neurosci Lett. 2014;558:159–63. 10.1016/j.neulet.2013.11.006 . 24246904

[40] Bennett-Guerrero E, Swaminathan M, Grigore AM, Roach GW, Aberle LG, Johnston JM, et al A phase II multicenter double-blind placebo-controlled study of ethyl pyruvate in high-risk patients undergoing cardiac surgery with cardiopulmonary bypass. J Cardiothorac Vasc Anesth. 2009;23(3):324–9. 10.1053/j.jvca.2008.08.005 . 18835526

